# Simulation and Experimental Study of the Multisized Silver Nanoparticles Sintering Process Based on Molecular Dynamics

**DOI:** 10.3390/nano12061030

**Published:** 2022-03-21

**Authors:** Mingfei Gu, Tingting Liu, Xingzhi Xiao, Gang Li, Wenhe Liao

**Affiliations:** School of Mechanical Engineering, Nanjing University of Science and Technology, Nanjing 210094, China; mingfei@njust.edu.cn (M.G.); ligang@njust.edu.cn (G.L.); cnwho@njust.edu.cn (W.L.)

**Keywords:** electronic materials, multisized nanoparticles, sintering behavior, molecular dynamics

## Abstract

Multisized nanoparticles (MPs) are widely employed as electronic materials to form conductive patterns, benefitting from their excellent sintering properties and mechanical reliability. However, due to the lack of effective detection methods for the real-time sintering process, it is difficult to reveal the sintering behavior during the MPs sintering process. In this work, a molecular dynamics method is used to track the trajectory of silver atoms. The melting behavior of a single nanoparticle (SP) is first discussed. The structural evolution of equally sized nanoparticles (EPs) and unequally sized nanoparticles (UPs) during the sintering process is analyzed alongside morphology changes. It is proposed that the UPs sintering process benefits from the wetting behavior of small-sized nanoparticles on the surface of large-sized nanoparticles, and the sintering angle (θ) is proposed as an index to estimate the sintering result of UPs. Based on the works above, three basic sintering modes and one advanced sintering mode in the MP sintering process are analyzed emphatically in this paper, and the roles of different-sized nanoparticles in MPs are concluded from simulation and experimental results. This work provides theoretical support for conductive ink composition design and sintering process optimization.

## 1. Introduction

Silver nanoparticles have been widely utilized as conductive components to form conductive patterns through sintering [1,2], which is attributed to their unique thermal [3], electrical [4], and mechanical properties [5]. However, single-sized nanoparticles commonly suffer from a low initial stacking density, which is only approximately 55–65% of bulk silver [6]. To obtain a high initial stacking density, multisized-nanoparticles (MPs) have been proposed as a promising replacement by mixing different-sized nanoparticles together [7], and filling the interspace between large-sized nanoparticles with small-sized nanoparticles [8].

The heat-based sintering of silver nanoparticles is a crucial metallurgical method for the fabrication of nanomaterials with tailored properties and is frequently utilized in academic research and industrial production [9,10]. The surface curvature and surface energy of silver nanoparticles are much higher than those of "bulk" silver, which is beneficial to the sintering of nanoparticles and makes the sintering process different from that of micro/milli-meter silver particles [11].

Numerous studies have been conducted to reveal the sintering process of nanoparticles. The initial simulation design configuration and associated melting temperature have been proven to exert considerable influence over sintering dynamics and thermal stability [12]. Buesser et al. extracted the sintering rate of nanoparticles from the simulation, which showed that lower temperatures or larger initial particle diameters led to slower sintering [13]. The governing sintering mechanism was found to be a variety of plasticity mechanisms, including dislocation, twinning, and amorphization, due to the high stress levels near the neck regions [14]. In addition, a broad range of possible interaction mechanisms were observed, from simple nanoparticle reorientation to atomic adsorption, neck formation, twinning within the nanoparticles, and full consolidation into a single and larger nanoparticle [15,16].

Different nanoparticles sintering mechanisms have been proposed. The atomistic simulation results revealed that pre-melting on the surface layer, which gives low activation energy and mechanistic behavior, appears similar to viscous flow [17]. Large variations in particle neck growth and shrinkage rates are considered as a function of grain boundary type, and faster sintering rates result from increased grain boundary misorientation angles [18,19]. Seong proposed that crystalline misalignment between the crystal structures results in enhanced grain boundary diffusion [20]. Dislocation evolution was also observed in the nanoparticle sintering process [21]. A general consensus has been reached that the sintering of silver nanoparticles is mainly carried out by surface diffusion and grain boundary diffusion among various sintering mechanisms, including stress-induced plasticity, elastic deformation, and surface diffusion [15,22]. However, these works have mainly focused on the equally sized nanoparticles sintering process, while the sintering mechanism of MPs is not yet clear. The different sintering behaviors can be observed obviously for different-sized nanoparticles. Therefore, it is expected that the dominant sintering mechanism is also different.

Molecular dynamics (MD) allows tracking of the real-time detailed motion of atoms and can provide valuable insight into the early stages of the nanoparticle sintering process, breaking through experimental observation limitations [23]. In this work, MD is employed to simulate the sintering process of MPs. The temperature and size effects on sintering are investigated by the evolution of shrinkage (ζ), local crystalline structure, and potential energy during sintering. The roles of different-sized nanoparticles in MPs are concluded from the MPs sintering simulation and experimental results, which further provides theoretical support for composition design and sintering process optimization.

## 2. Materials and Methods

### 2.1. Molecular Dynamics Simulation Implementation

The embedded atom (EAM) potential [24] has been used successfully to investigate Face-Centered Cubic (FCC)-based noble metal properties, accurately reproducing lattice parameters, cohesive energy, elastic constants, and phonon frequencies [25]. In this work, an existing EAM potential [26] was employed as the force field to describe the interactions between silver atoms in our simulation. In EAM, the energy of the system is given by

(1) $$E_{tot}=\frac{1}{2}\underset{ij}{\sum }V_{ij}\left(r_{ij}\right)+\underset{i}{\sum }F_{i}\left({\bar{\rho }}_{i}\right)$$

where *V_ij_* stands for the pairwise interaction energy between atoms *i* and *j* separated by distance *r_ij_*, *F_i_* stands for the embedding energy of atom *i*, and ${\bar{\rho }}_{i}$ stands for the electron density calculated by density functional theory.

Modeling and simulation were performed using the open-source Large-scale Atomic/Molecular Massively Parallel Simulator (LAMMPS) code [27]. The NVT (numbers, volume, and temperature are constant) canonical ensemble with the Nose–Hoover thermostat [28,29] was employed to control the temperature in the simulation. The time step was set as 0.001 ps. The trajectory of silver atoms was visualized in OVITO software, which is also open-source [30].

### 2.2. Modeling and Heating Settings

A cubic simulation box with shrink-wrapped boundaries was first built, which is an isolated system, and silver atoms were created inside the box to obtain a large cubic FCC crystal. Spherical silver nanoparticles with different particle diameters were extracted from crystals. These FCC nanoparticles were initially perfect spheres, and energy minimization of these nanoparticles was performed to obtain an optimal initial structure. These nanoparticles were equilibrated at 300 K for 10 ps. Four different models of silver nanoparticles were established, including a single nanoparticle (SP), equally sized nanoparticles (EPs), unequally sized nanoparticles (UPs), and multisized nanoparticles (MPs), whose parameters are listed in Table 1.

Five nanoseconds of melting simulation in 5,000,000 steps were taken to heat the SP from 300 K to 1400 K at a heating rate of 0.22 K per picosecond, which is a sufficient timescale to obtain the actual motion of atoms. The duration of the sintering simulation was 500 ps in 500,000 steps. Although an actual sintering process is longer than this timescale, 500 ps is enough to observe early-stage sintering behavior [31]. The isothermal heating (IH) method was employed, thus eliminating the temperature gradient inside the nanoparticles during the sintering process. EPs were heated at temperatures below and above the melting point of the nanoparticles. UPs and MPs ware heated at 1000 K to study the different sintering behaviors of nanoparticles with different sizes in one sintering process.

### 2.3. Analysis Method of Simulation Results

The atomic potential energy of silver nanoparticles during the melting process was calculated, and the temperature at which the atomic potential energy increased sharply was regarded as the melting point [32,33]. The atomic potential energy per atom instead of atomic potential energy was used in this work to better understand the size effects on the thermal stability of silver nanoparticles.

The shrinkage coefficient (*ζ*) was used to identify the different sintering stages of silver nanoparticles [34]. *ζ* is the ratio of the change in distance to the initial distance, which is given by

(2) $$\zeta =\frac{\Delta L}{L}=\frac{L_{0}-L}{L_{0}}$$

where *L*_0_ stands for the distance of the centers of mass when the simulation begins, and *L* stands for the instantaneous distance during the sintering process.

Common neighbor analysis (CNA) was employed to analyze the local structural evolution during the heating process [35,36,37]. CNA describes the stacking structure of atoms through the bonding relationship between two atoms and the surrounding shared atoms. When the distance between two atoms is less than the nearest neighbor distance (*r_cut_*) determined by the first peak and valley of the radial distribution function, the two atoms are bonded. For FCC structures, $r_{cut}^{FCC}$ is given by

(3) $$r_{cut}^{FCC}=\frac{a_{FCC}\left(1/2^{0.5}+1\right)}{2}\approx 0.854a_{FCC}$$

where $a_{FCC}$ stands for the lattice constant of the FCC crystal structure. The obtained cut-off radius for silver is 3.491, according to Equation (3). Local orders of atoms are classified into three categories: (1) face-centered-cubic (atoms in a local FCC order), (2) hexagonal closed packed (atoms in a local HCP order), and (3) amorphous (atoms in all other local orders).

### 2.4. Sintering Experiment and Microstructure Characterization

The MPs were composed of 2 g of 80 nm nanoparticles and 2 g of 20 nm nanoparticles with a weight ratio set to 1:1, both of which were purchased from Hongwu International Group Ltd., Guangzhou, China. The MPs were coated on a cleaned Cu substrate and sintered at 1150 K for 10 min to prepare the sample. The morphology of the sintered MPs sample was observed by scanning electron microscopy (GeminiSEM 300, ZEISS, Jean, Germany).

## 3. Results and Discussion

### 3.1. Size-Dependent Thermal Stability and Temperature Dependence

**Figure 1:**
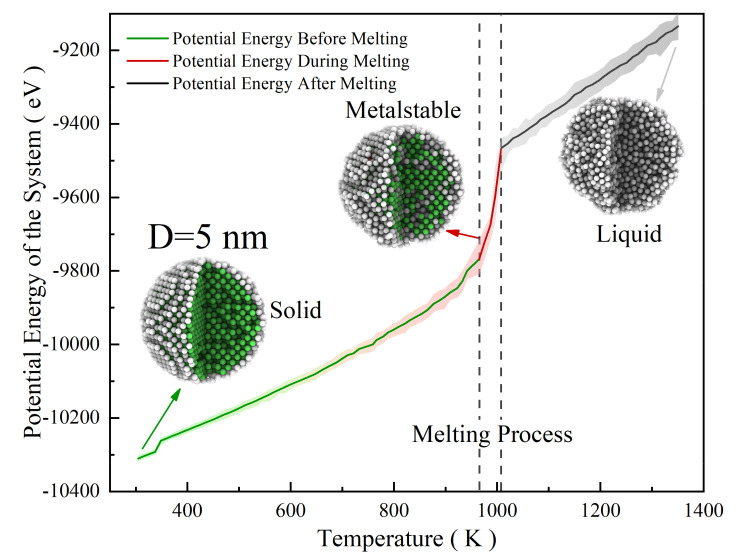
Potential energy of SP-5 as a function of heating temperature.

Melting behavior during the single nanoparticle melting process: Thermal stabilities of the SP with different sizes are studied in this section. The potential energy (PE) of the single nanoparticle (SP) in the temperature range from 300 K to 1400 K is calculated. The PE increases linearly with temperature before melting, and when the temperature increases to a specific value, the PE/atom shows a step response, indicating that surface premelting occurs, corresponding to the nanoparticle melting point (Figure 1, Movie S1).

**Figure 2:**
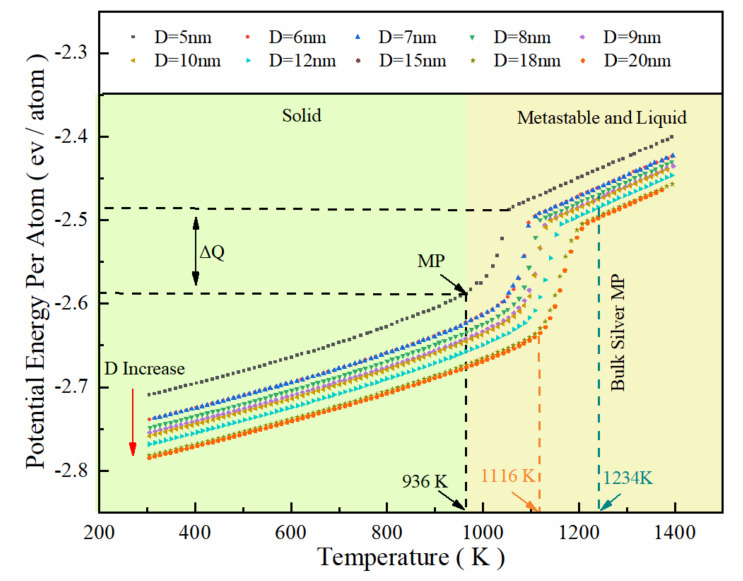
Potential energy per atom of SP with different diameters (from 5 nm to 20 nm) as a function of heating temperature with lines in different colors, alongside the corresponding regions for solid (green), metastable, and liquid (yellow).

The melting point of silver nanoparticles is much lower than that of bulk silver (1234 K), according to the potential energy per atom (PE/Atom) of SP with diameters of 5 nm to 20 nm (Figure 2). The melting point of SP-5 (SP with a diameter of 5 nm) is 936 K, while that of SP-20 (SP with a diameter of 20 nm) is 1159 K, indicating that the melting point decreases with decreasing particle size. Due to the decrease in the particle size, the specific surface energy of SP increases sharply with the specific surface area. The SP exhibits high activity and instability; thus, a lower energy is required to break the unstable structure, reflecting the size dependence of thermal stability.

This size effect can be further expressed by reciprocals of the diameter, given by

(4) $$T_{m}=T_{\mathrm{mb}}-T_{\mathrm{mb}}\frac{\beta }{D}$$

where Tm stands for the melting point of SP, Tmb stands for the melting point of bulk metal, β stands for the coefficient depending on the material category, and D stands for the diameter of SP. Based on the best fitting line to our MD result, the melting point of SP is given by

(5) $$T_{m}=1192-\frac{1164}{D}$$

**Figure 3:**
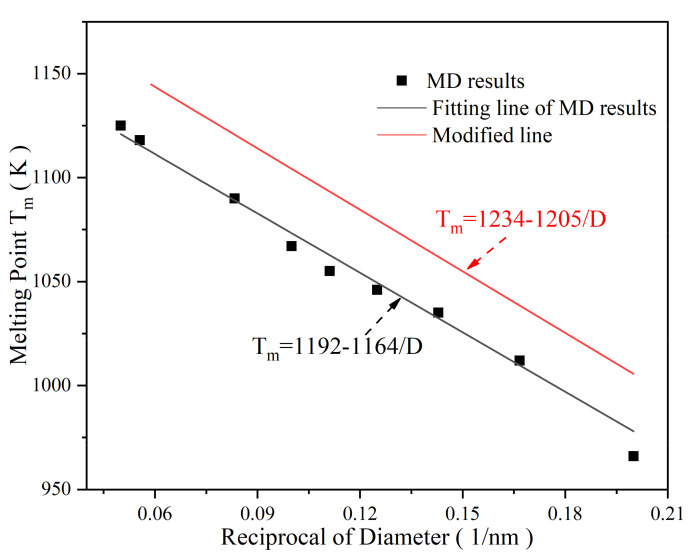
Melting point of nanoparticles as a function of the reciprocal of diameter.

The melting point of bulk silver is 1192 K by setting the diameter to be infinitely large from Equation (5), which is close to the actual value of 1234 K with an error of 3.40%. Equation (5) is modified [38] with the bulk silver melting point in Figure 3 as

(6) $$T_{m}=1234-\frac{1205}{D}$$

**Figure 4:**
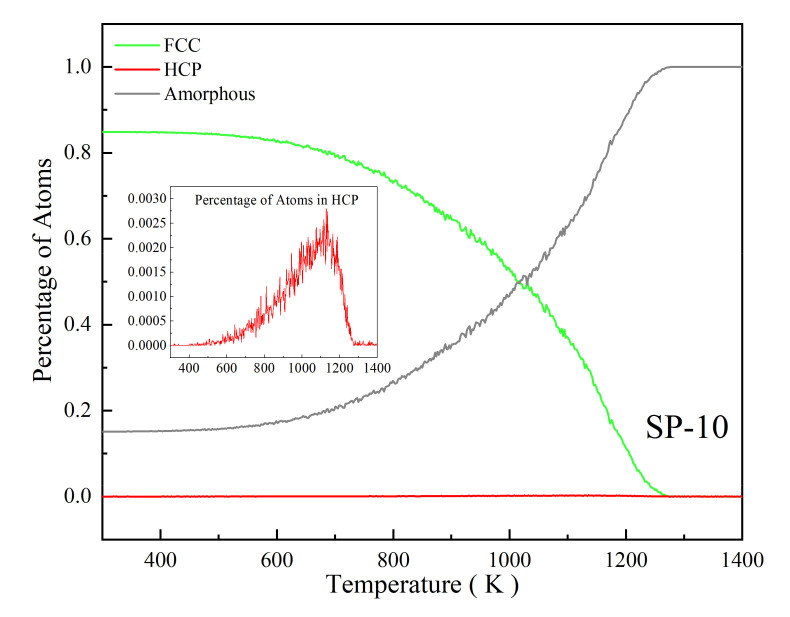
Percentage of atoms in different local structures corresponding to temperature.

**Figure 5:**
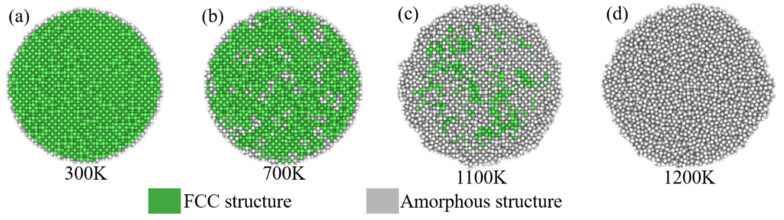
Structural evolution of SP-10 during the melting process heating from 300 K to 1200 K (**a**–**d**).

The percentage of atoms in different local structures of all atoms corresponding to temperature is shown in Figure 4, indicating that the structural evolution during SP melting is a process in which the ordered FCC structure transforms into an amorphous structure, and this transformation depends on temperature. Figure 5 shows the structural evolution of SP-10 (whose melting point is 1114 K calculated from Equation (6)) during the melting process. The atoms are in an ordered FCC structure at room temperature (300 K, Figure 5a). At a temperature far below the MP (700 K, Figure 5b), most atoms remain in order, and the SP remains rigid. When the temperature rises close to the melting point (1100 K, Figure 5c), the surface atoms change amorphously, while some internal atoms remain in the FCC order, indicating that the SP is premelting. After the temperature rises above the melting point (1200 K, Figure 5d), the atoms are highly disordered and completely transform into an amorphous state, which means that the SP completely melts and is in the liquid phase.

### 3.2. Sintering Stages and Structural Evolution during the Equally Sized Nanoparticle Sintering Process

**Figure 6:**
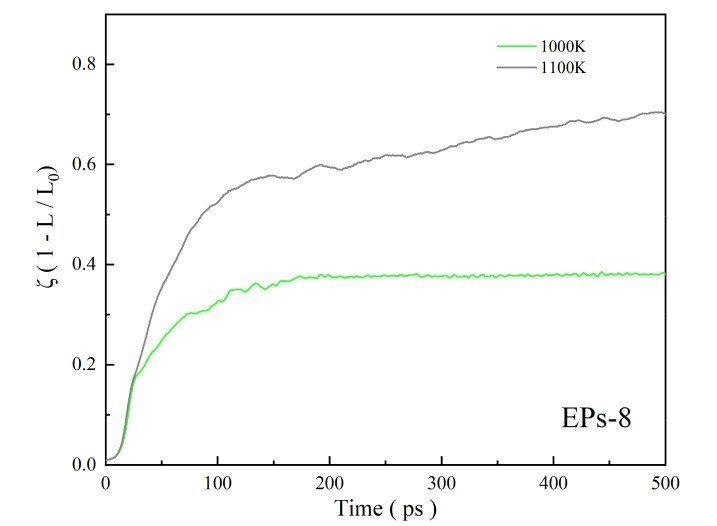
Shrinkage (ζ) of equally sized nanoparticles with a diameter of 8 nm during surface premelting sintering and liquid-phase sintering.

**Figure 7:**
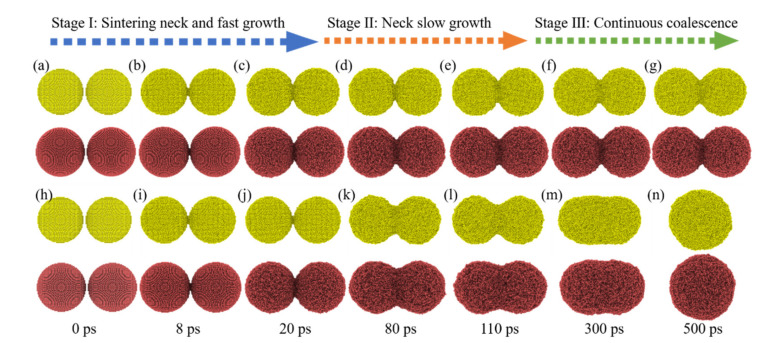
EPs morphological changes during the sintering process corresponding to sintering time. (**a**–**g**) Premelting sintering process (sintering temperature is 1000 K) and (**h**–**n**) liquid-phase sintering process (sintering temperature is 1100 K). The EPs-8 atoms are colored yellow and EPs-12 atoms are colored red.

To analyze the sintering mechanism of equally sized nanoparticles (EPs) under different conditions, EPs with varying particle size in the range from 5 nm to 20 nm are heated at temperatures below and above the melting point, calculated from the proposed model in Section 3.1. EPs-8 (8 nm nanoparticle whose melting point is 1084 K) sintered at 1000 K (Movie S2) and 1100 K (Movie S3) and EPs-12 (12 nm nanoparticle whose melting point is 1134 K) sintered at 1100 K and 1200 K are discussed as representatives. Figure 6 shows that the sintering process of EPs can be divided into three stages according to the shrinkage of nanoparticles mass centers with sintering time. (1) Stage Ⅰ: a sintering neck forms and grows fast; (2) Stage Ⅱ: the sintering neck grows slowly; (3) Stage Ⅲ: the nanoparticles continuously coalesce. In Stage Ⅰ (0–20 ps), shrinkage is independent of temperature, and ζ increases from 0 to 0.2 within 20 ps, indicating that nanoparticles approach each other and shrink rapidly, so the sintering behavior of EPs in this stage is sintering neck formation and fast growth, as shown in Figure 7a–c,h–j. In Stage Ⅱ (20–110 ps), shrinkage depends on temperature. ζ increases from 0.2 to 0.35 at 1000 K (below melting point) and increases from 0.2 to 0.6 at 1100 K (above melting point) within 90 ps, which means that the shrinkage intensifies with temperature. ζ shows a significantly slower growth rate in Stage Ⅱ than in Stage Ⅰ, indicating that the sintering behavior of EPs in Stage Ⅱ is slow sintering neck growth, as shown in Figure 7c–e,j–l. Stage Ⅲ (110–500 ps) is the nanoparticle continuous coalescence stage. When the sintering temperature is lower than melting point, ζ is stable at approximately 0.4, indicating that the nanoparticles no longer shrink, as shown in Figure 7e–g. When the sintering temperature is above than melting point, ζ increases slowly from 0.6 to 0.7 within 390 ps, during which two nanoparticles coalesce into one new, large particle, as shown in Figure 7l–n. It can also be found that EPs composed of nanoparticles with different particle sizes show the similar sintering process.

**Figure 8:**
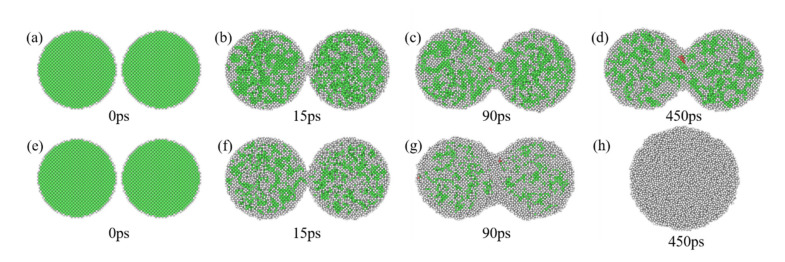
The structural evolution of EPs-8 during the sintering process corresponding to the sintering time. (**a**–**d**) Premelting sintering process (sintering temperature is 1000 K) and (**e**–**h**) liquid-phase sintering process (sintering temperature is 1100 K). The color bar is the same as that in Figure 5.

**Figure 9:**
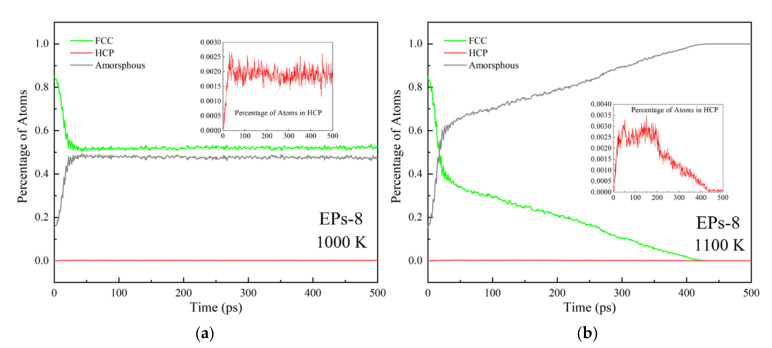
The percentage of atoms in different local structures corresponding to temperature during the premelting sintering process (**a**) and the liquid-phase sintering process (**b**).

Figure 8 and Figure 9 show the structural evolution during the EPs-8 sintering process. Premelting sintering occurs when the sintering temperature is 1000 K, as shown in Figure 8a–d. The formation and growth of the sintering neck can be observed, and the neck does not continue to grow after growing to a specific size. Finally, a stable sintered body is formed. A small amount of HCP structure can be found at the sintering neck during this process, indicating that layer faults are generated in this region. Figure 9a shows that the structure of nanoparticles rapidly transforms from FCC to HCP and amorphous in 20 ps during premelting sintering, indicating that the structural evolution completes within 20 ps and few structural evolutions occur after that.

Liquid-phase sintering occurs when the sintering temperature is 1100 K, as shown in Figure 8e–h, which shows a similar structural transformation to premelting sintering in the early stage. During the liquid-phase sintering process, FCC structures continue to transform into HCP and amorphous structures throughout the sintering process, and finally, all structures become amorphous. The structures transform rapidly from the original FCC structures to HCP and amorphous structures in the initial 20 ps, and after that, it takes approximately 400 ps (20–420 ps) for all the structures to transform slowly into amorphous structures, as shown in Figure 9b. Figure 8 and Figure 9 further indicate that the sintering stages depend on the sintering time, and the structural evolution depends on the sintering temperature during the EP sintering process.

### 3.3. Wetting Behavior of Small-Sized Nanoparticles during the Unequally Sized Nanoparticle Sintering Process

**Figure 10:**
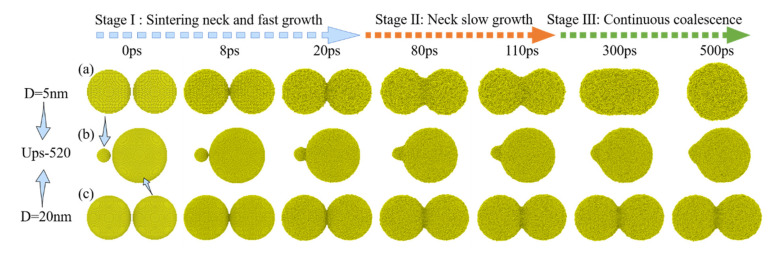
Morphology changes of different models during the sintering process corresponding to the sintering time, all of which are sintered at the same temperature (1000 K). (**a**) EPs-5 sintering process, (**b**) UPs-520 sintering process, and (**c**) EPs-20 sintering process.

**Figure 11:**
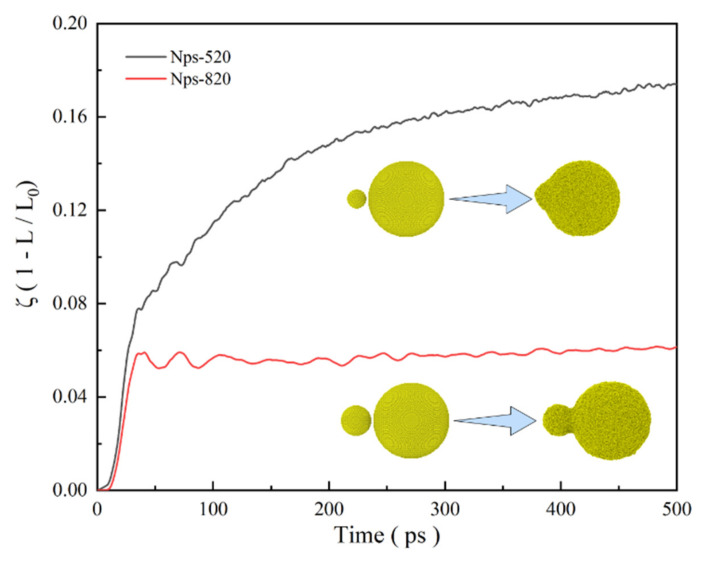
Shrinkage (ζ) of two equally sized nanoparticles during the sintering process with a sintering temperature between the UPs.

The surface morphology changes of UPs-520 (a 5 nm NP whose melting point is 993 K and a 20 nm NP whose melting point is 1174 K) sintered at 1000 K as an example of an unequally sized nanoparticle (UPs) sintering process are shown in Figure 10, and for comparison, the morphology changes of EPs-5 and EPs-20 sintered under the same conditions are also shown. The UPs sintering process also shows similar surface morphology changes to the EP sintering process, including sintering neck formation and fast growth, slow neck growth, and continuous coalescence. As the sintering temperature is between the melting point of small-sized nanoparticles and that of large-sized nanoparticles, small-sized nanoparticles show the characteristics of liquid-phase sintering, while large-sized nanoparticles show the characteristics of premelting sintering, causing the UPs sintering process to have both sintering behaviors at the same time. Figure 11 shows the shrinkage of the centers of mass of UPs during the sintering process, which is similar to the shrinkage of the EPs shown in Figure 6. This shrinkage of UPs is due to small-sized nanoparticles approaching large-sized nanoparticles and melting on the surface of large-sized nanoparticles, comparing Figure 10b to Figure 10a,c.

**Figure 12:**
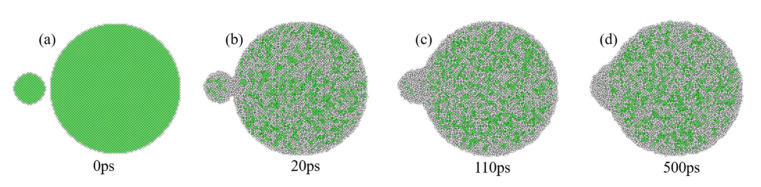
The structural evolution of UPs-520 during the sintering process corresponding to the sintering time when sintered at 1000 K when (**a**) 0 ps, (**b**) 20 ps, (**c**) 110 ps and (**d**) 500 ps. The color bar is the same as that in Figure 5.

The structural evolution of UPs during the sintering process also shows both premelting sintering and liquid-phase sintering characteristics. The local structures of atoms of small-sized nanoparticles change rapidly from FCC to amorphous, and all FCC structures disappear after sintering, consistent with the structural evolution of liquid-phase sintering. The structural evolution is consistent with the premelting sintering process, in which atoms partially transform from FCC to amorphous, as shown in Figure 12. It is also observed that some HCP structures are generated at the neck. The structural evolution indicates that the small-sized nanoparticles in UPs completely melt, while the large-sized nanoparticles remain rigid because of their different size-dependent MPs, while the sintering temperature is between the MPs of different-sized nanoparticles.

Small-sized nanoparticles approach large-sized nanoparticles and begin melting at the same time. The structural evolution of small-sized nanoparticles is divided into two stages (Figure 9b) with a significant time point of 20 ps. Before this time point, small-sized nanoparticles act more similar to solids because most atoms inside are in the FCC structure, while after 20 ps, amorphous structures dominate, and small-sized nanoparticles show more liquid characteristics. It takes approximately 8 ps for small-sized nanoparticles to approach large-sized nanoparticles (Figure 10b), indicating that UPs sinter under the premelting condition within 8 ps to 20 ps. After 20 ps, due to the small-sized nanoparticles being liquid-like and large-sized nanoparticles being solid-like, the sintering behavior of UPs is similar to the macroscopic wetting of a solid surface by a liquid droplet. As this wetting-like process takes the main percentage of sintering time, the sintering process of UPs can be regarded as a process in which small-sized nanoparticles wet large-sized nanoparticles.

**Figure 13:**
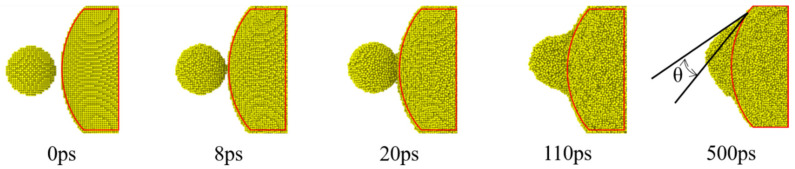
The sintering angle (*θ*), which is defined as the angle between the tangential line of the melting small-sized nanoparticle liquid surface and the tangential line of the rigid large-sized nanoparticle solid surface, forms and grows during the sintering process. The red line is the edge of large-sized nanoparticles.

Another difference between the UPs sintering process and EP sintering process is the final morphology of the sintering neck. In the EP sintering process, the final sintering neck grows to a specific size during premelting sintering (Figure 10c) or disappears during liquid-phase sintering (Figure 10a), while the final neck in UPs is not similar to either of these two kinds (Figure 10b). To describe the result of UP sintering, a “sintering angle” (*θ*) similar to the wetting angle is suggested in this work, which is defined as the angle between the tangential line of the melting small-sized nanoparticle liquid surface and the tangential line of the rigid large-sized nanoparticle solid surface, as shown in Figure 13. A smaller sintering angle indicates that small-sized nanoparticles wet large-sized nanoparticles better, and there is also better fusion of the sintered silver body.

**Figure 14:**
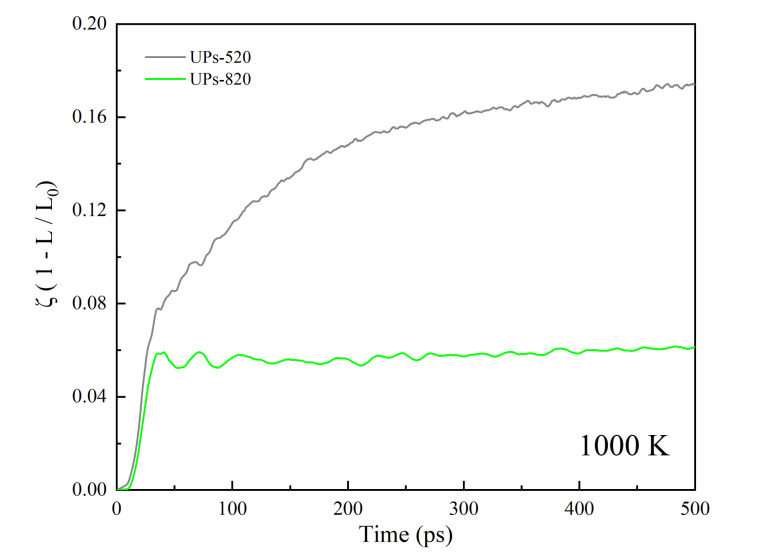
Shrinkage (ζ) of different UPs sintered at 1000 K corresponding to the sintering time.

How the size difference of nanoparticles affects the sintering results of UPs is discussed by changing the small-sized nanoparticle size and showing the shrinkage of the centers of mass of UPs-520 and UPs-820 (8 nm nanoparticles and 20 nm nanoparticles) during sintering at 1000 K (Movie S4) in Figure 14. The shrinkage of UPs-820 is not as substantial as that of UPs-520, with ζ being stable at approximately 0.06, which is much lower than that of UPs-520. This consequence results from the different sintering mechanisms during these two UP sintering processes determined by temperature. As the sintering temperature (1000 K) is higher than the melting point of SP-5 (993 K, calculated from Equation (6)) but lower than that of SP-8 (1084 K, calculated from Equation (6)), the UPs-520 sintering behavior is similar to that of EPs liquid-phase sintering, while the UPs-820 sintering behavior is similar to that of EPs premelting sintering.

### 3.4. Different Sintering Behaviors Determined by Nanoparticle Sizes in the Multisized Nanoparticle Sintering Process

**Figure 15:**
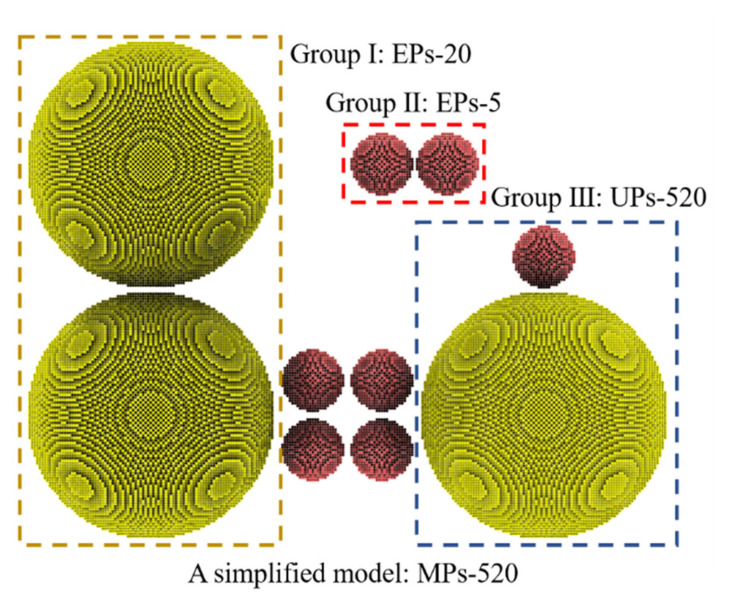
A simplified model MPs-520 was used to represent the composition of MPs, including three basic groups. Nanoparticles with diameters of 5 nm are colored red, and those with diameters of 20 nm are colored yellow.

Multisized nanoparticles (MPs) can be divided into several basic nanoparticle groups according to the particle sizes of two adjacent nanoparticles. In this work, a simplified MPs model was constructed to describe the complex sintering behavior of MPs. The model (MPs-520, Figure 15) is simplified based on two rules: (1) only two different sizes of nanoparticles are used, 5 nm and 20 nm; (2) the edge-to-edge distance of each pair of nanoparticles is set to 0.5 nm. It can be easily observed that MPs-520 comprises three groups, including EPs-20, EPs-5, and UPs-520. These three groups are expected to represent the three basic nanoparticles groups in MPs: (1) Small-sized EPs: a pair of generalized “equally small-sized” nanoparticles referring to all nanoparticles whose melting points are below the heating temperature. (2) Large-sized EPs: a pair of generalized “equally large-sized” nanoparticles referring to all nanoparticles whose melting points are over the heating temperature. (3) UPs: a pair of “unequally sized” nanoparticles, i.e., a pair of nanoparticles whose melting points are below and over the heating temperature, respectively.

**Figure 16:**
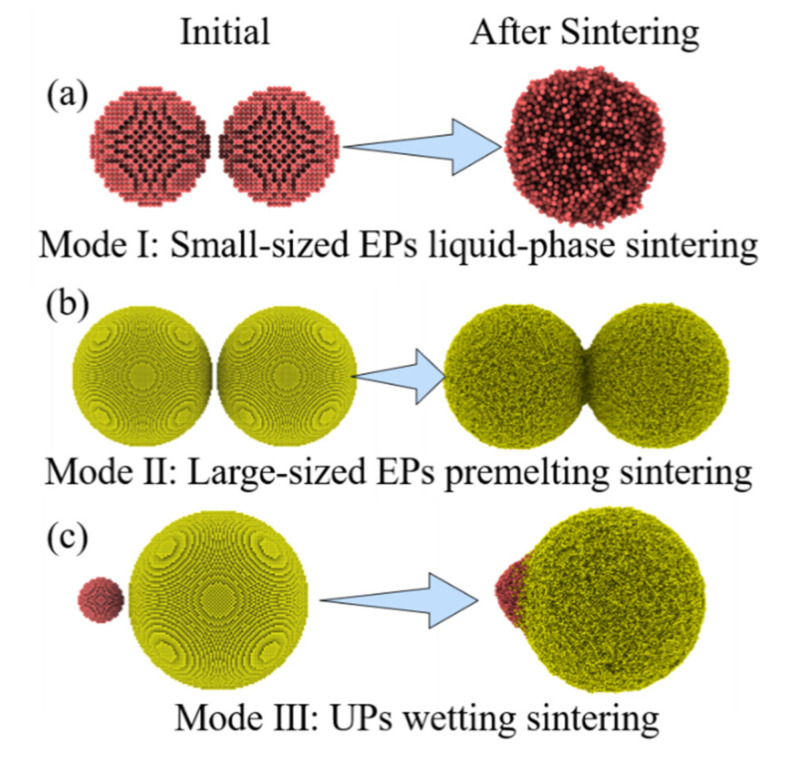
(**a**–**c**) Three basic sintering modes during the MP sintering process.

These three basic groups have their own sintering behaviors during the MPs sintering process, which are explained by the MPs-520 sintering behavior heated at 1000 K in this work. The sintering process of EPs-5 (whose melting point is 993 K) is used to represent that of small-sized EPs, the sintering process of EPs-20 (whose melting point is 1174 K) is used to represent that of large-sized EPs, and the sintering process of UPs-520 represents that of UPs, as shown in Figure 16. When heated at 1000 K, EPs-5 exhibits a typical EP liquid-phase sintering process and aggregates into new and larger nanoparticles after sintering. EPs-20 shows a typical EP premelting sintering process, forming a sintered body with a stable sintering neck after sintering. UPs-520 exhibits a typical wetting sintering process in which 5 nm silver particles form a stable wetting structure on the surface of 20 nm silver particles. Therefore, the MPs sintering process can be classified into three basic modes: (1) liquid-phase sintering of small-sized EPs; (2) premelting sintering of large-sized EPs; and (3) wetting sintering of UPs.

**Figure 17:**
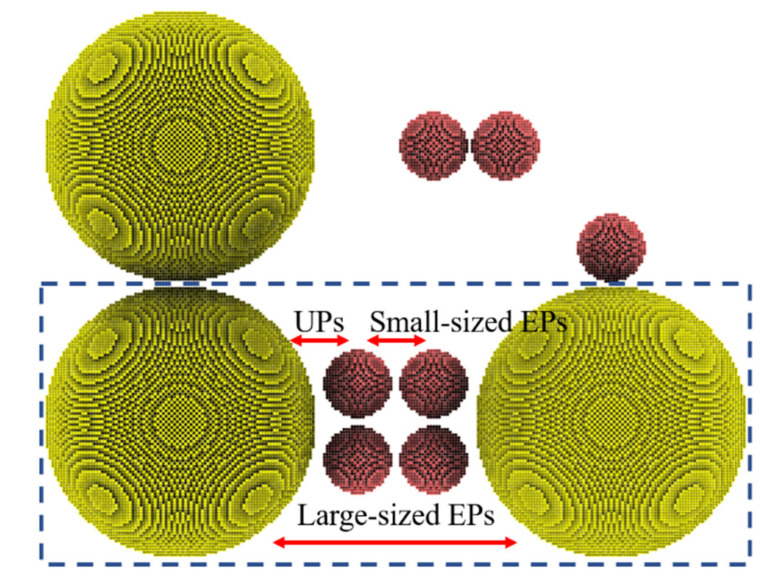
UPs-like local structures in MPs, in which large amounts of small EPs dope among large EPs.

**Figure 18:**
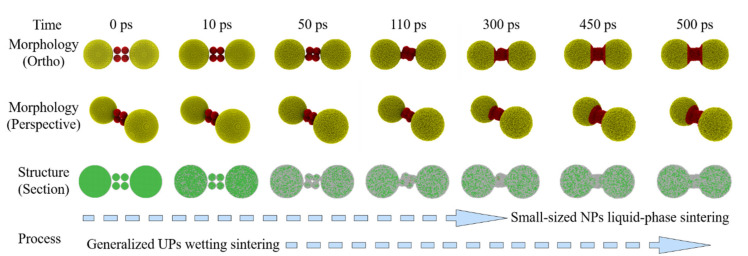
Sintering process for a simplified-model MPs-520, which represents the UPs-like structure in MPs.

**Figure 19:**
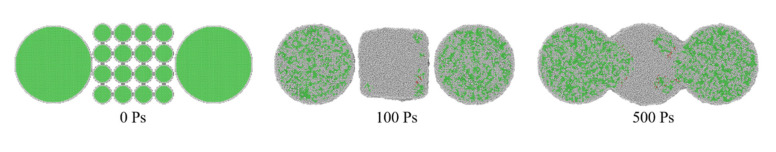
Sintering process of new MPs-520.

Taking a further analysis, the sintering unit of MPs is composed of these three basic groups, and thus, the sintering process shows an advanced sintering mode. In MPs, large amounts of small-sized EPs dope among large-sized EPs and form complex UPs-like local structures with different numbers of these three groups. Based on the understanding of the three basic sintering modes, the sintering behavior of this UPs-like structure can be analyzed by modeling a simplified model composed of two large nanoparticles (20 nm) and several small nanoparticles (5 nm), as shown in Figure 17. Figure 18 shows the sintering behavior of this model. Liquid-phase sintering of small-sized nanoparticles first takes place during the MPs sintering process, in which small nanoparticles coalesce into new nanoparticles, and this liquid-phase sintering continues throughout the sintering process. Once the new NP touches the edge of large-sized nanoparticles, UP wetting sintering begins. This wetting sintering is more generalized than that discussed above in two aspects. The first difference is the particle size. It has been discussed that the wetting behavior in UPs is based on the melting point difference caused by size differences; more specifically, wetting takes place between small-sized nanoparticles and large-sized nanoparticles. However, during the MPs sintering process, the new nanoparticles coalesced by small-sized EP liquid-phase sintering take the role of single small-sized nanoparticles in the UPs, while their particle size may not be limited. The particle size of the new nanoparticles depends on the number of original small-sized EPs coalesced and may be smaller, equal to, or larger than that of the original large-sized nanoparticles, which does not change the role of these nanoparticles. Another difference is the sintering behavior of these new nanoparticles when their particle size is large. For example, during the three EPs-20 (three 20 nm nanoparticles) sintering process at 1000 K, all of the nanoparticles obviously show the characteristics of premelting sintering. Replacing the middle SP-20 with 64 5 nm nanoparticles to make up a new MPs-520 (Movie S5) the wetting process of these MPs shows the characteristics of wetting sintering instead of premelting sintering because of the total melting of the new large-sized nanoparticles, as shown in Figure 19.

**Figure 20:**
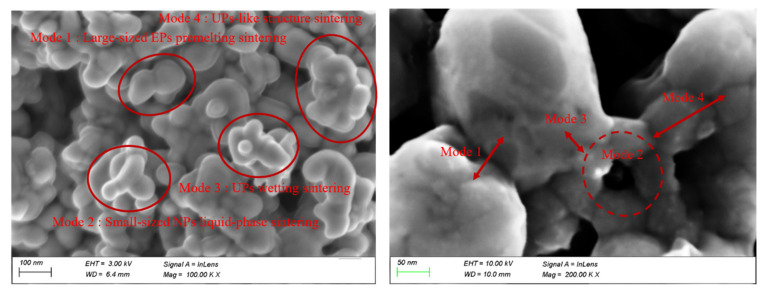
SEM image of the sintered MPs microstructure, which shows the three basic sintering modes and one advanced sintering mode.

The sintering behavior of MPs can thus be explained by these three basic sintering modes and one advanced sintering mode, including small-sized EP liquid-phase sintering, large-sized EP premelting sintering, UP wetting sintering, and UPs-like structure sintering, which is consistent with the sintering experiment result in Figure 20. It is also observed and concluded that in a reasonably designed and well-prepared MPs sintering process, UPs-like structure sintering plays the most important role, while the three basic modes should be avoided. The sintering behavior reveals the role of different-sized nanoparticles in MPs; that is, large-sized nanoparticles are mainly responsible for reducing porosity and providing structural rigidity in the silver sintering body, while small-sized nanoparticles are melted and sintered as adhesives, combining large-sized nanoparticles together to provide muscular bonding strength and achieve the purpose of sintering.

## 4. Conclusions

To clarify the structural evolution and sintering behavior of multisized nanoparticles, the sintering processes of four different models of silver nanoparticles (SP, EPs, UPs, and MPs) were studied by MD simulation. According to the simulation and experimental results, the following conclusions can be obtained:

(1) The sintering process of EPs can be divided into three stages: sintering neck formation and fast growth, slow neck growth, and continuous coalescence.
(2) During the sintering process of UPs, small-sized nanoparticles melt, move to the surface of the more significant nanoparticles quickly, and wet the surface of large-sized nanoparticles. This wetting behavior becomes clearer with the increase in size difference between the two nanoparticles.
(3) The sintering behavior of MPs is composed of three basic sintering modes and one advanced sintering mode, including small-sized EPs liquid-phase sintering, large-sized EPs premelting sintering, UPs wetting sintering, and UPs-like structure sintering.
(4) After sintering, large-sized nanoparticles nearly retain their initial shapes and are macroscopically connected by small-sized nanoparticles to form a sintered body.

## Figures and Tables

**Table 1:** Configuration of the simulation models in this work.

Model	SP	EPs	UPs	MPs
Diameter (nm)	5–20	Equally sized	Unequally sized	Multisized
Edge-to-edge distance (nm)	-	0.5	0.5	0.5
Atom numbers	3805–244,961	7610–489,922	248,766; 260,644	505,142
Morphology ^1^	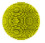	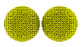	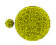	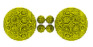

^1^ The silver atoms are colored yellow.

## Data Availability

Not applicable.

## Supplementary Materials

The following supporting information can be downloaded at: https://www.mdpi.com/article/10.3390/nano12061030/s1, Movie S1: Melting process of 5 nm single nanoparticle (.AVI); Movie S2: EPs premelting sintering process (.AVI); Movie S3: EPs liquid-phase sintering process (.AVI); Movie S4: UPs wetting sintering process (.AVI); Movie S5: MPs sintering process (.AVI).
